# The Physiology of Insulin Clearance

**DOI:** 10.3390/ijms23031826

**Published:** 2022-02-05

**Authors:** Richard N. Bergman, Morvarid Kabir, Marilyn Ader

**Affiliations:** Diabetes and Obesity Research Institute, Cedars-Sinai Medical Center, Los Angeles, CA 90048, USA; morvarid.kabir@cshs.org (M.K.); marilyn.ader@cshs.org (M.A.)

**Keywords:** insulin clearance, type 2 diabetes, beta-cell failure, disposition index

## Abstract

In the 1950’s, Dr. I. Arthur Mirsky first recognized the possible importance of insulin degradation changes to the pathogenesis of type 2 diabetes. While this mechanism was ignored for decades, insulin degradation is now being recognized as a possible factor in diabetes risk. After Mirsky, the relative importance of defects in insulin release and insulin resistance were recognized as risk factors. The hyperbolic relationship between secretion and sensitivity was introduced, as was the relationship between them, as expressed as the disposition index (DI). The DI was shown to be affected by environmental and genetic factors, and it was shown to be differentiated among ethnic groups. However, the importance of differences in insulin degradation (clearance) on the disposition index relationship remains to be clarified. Direct measure of insulin clearance revealed it to be highly variable among even normal individuals, and to be affected by fat feeding and other physiologic factors. Insulin clearance is relatively lower in ethnic groups at high risk for diabetes such as African Americans and Hispanic Americans, compared to European Americans. These differences exist even for young children. Two possible mechanisms have been proposed for the importance of insulin clearance for diabetes risk: in one concept, insulin resistance per se leads to reduced clearance and diabetes risk. In a second and new concept, reduced degradation is a primary factor leading to diabetes risk, such that lower clearance (resulting from genetics or environment) leads to systemic hyperinsulinemia, insulin resistance, and beta-cell stress. Recent data by Chang and colleagues appear to support this latter hypothesis in Native Americans. The importance of insulin clearance as a risk factor for metabolic disease is becoming recognized and may be treatable.

## 1. Introduction

Personal Note

In 1965, I graduated in engineering from the Case Institute of Technology (now Case Western Reserve University) and realized that I did not want to sit in a large room with a slide rule, a T-square and a protractor and be an electrical engineer. My uncle, Oscar Hechter, suggested I try physiology. I did not even know how to spell it, but I matriculated to a PhD program in physiology at the University of Pittsburgh Medical School. John Urquhart was modeling adrenocortical kinetics, and it seemed reasonable for me to enter his program and study cortisol turnover. However, as luck would have it, I was invited to work with I. Arthur Mirsky, a Professor in the Neuropsychiatric Institute. Professor Mirsky was a polymath, who did protein biochemistry in the morning, metabolic physiology in the afternoon, and practiced as a psychoanalyst in the evenings (fortunately, he never asked me to enter his psychoanalytic program). Dr. Mirsky was interested in insulin degradation [1,2,3,4,5]. Thus, it is a remarkable set of circumstances that have led our laboratory to return to insulin degradation as a possible pathogenic factor in type 2 diabetes. Arthur Mirsky was without question ahead of his time regarding the latter issue, and I only wish he were still around to enjoy the renewed interest in his work. Note that others have also rediscovered the monumental contributions of Arthur Mirsky [6].

## 2. Insulin Resistance and Insulin Secretion

**Figure 1:**
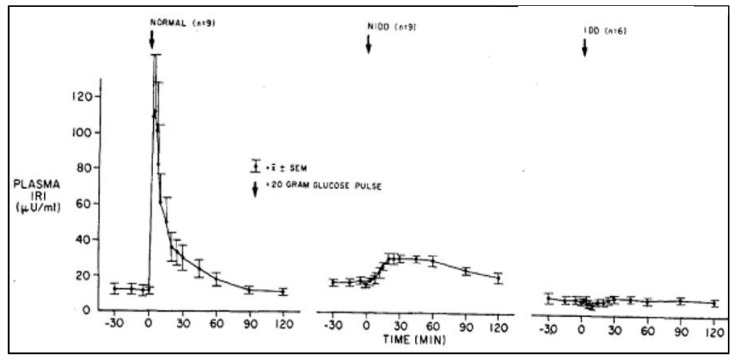
Insulin release (Plasma IRI: immunoreactive insulin) in response to the intravenous administration of glucose in normal and diabetic subjects. Mean fasting plasma glucose concentrations: normal subjects = 85 ± 3 mg/dL; noninsulin-dependent diabetic subjects (NIDDs) = 180 ± 10 mg/dL; and insulin-dependent diabetic subjects (IDDs) = 325 ± 33 mg/dL. Note the lack of first-phase insulin response and the preservation of second-phase insulin response in noninsulin-dependent diabetic subjects, and the total lack of any response to glucose in the insulin-dependent diabetic subjects. Pfeifer MA et al. AM J Med 70:579, 1981.

Insulin degradation was not a popular subject during Mirsky’s time, as the discovery of the radioimmunoassay by Yalow and Berson had focused attention on insulin secretory dysfunction as a very important factor in the pathogenesis of type 2 diabetes [7,8,9,10]. Daniel Porte and his colleagues demonstrated defects in insulin release in mild type 2 diabetics (Figure 1) [11].

Alternatively, Gerald Reaven confirmed the work of Himsworth, demonstrating relative insulin resistance in type 2 diabetes [12]. The importance of insulin resistance in disease pathogenesis lagged behind insulin response, as resistance was more difficult to measure than secretion. A similar lesson can be learned for insulin clearance, which itself is difficult to measure [13]; therefore, its importance is only being appreciated more recently. In fact, there was limited interest in insulin clearance as a pathogenic factor until recent years. Workers such as Sonia Najjar, editor of this series, have done outstanding work on the molecular events associated with insulin degradation [14,15,16]. Dr. Najjar et al. elucidated the specific role and the molecular mechanisms by which carcinoembryonic antigen-related cell adhesion molecule 1 (CEACAM1) promotes insulin clearance [17,18,19]. First studies showed that reduced CEACAM1 expression by mRNA transfection in H4-II-E rat hepatoma cells decreased receptor-mediated insulin internalization and degradation [15,20], and CEACAM1 phosphorylation regulates receptor-mediated insulin uptake and its degradation [15,21]. Liver-based rescuing of CEACAM1 restored insulin clearance, plasma insulin level, insulin sensitivity, and steatohepatitis caused by the global deletion of CEACAM1, and reversed the gain in body weight and total fat mass [22]. Mirsky and Broh-Kahn first discovered insulin degrading enzyme (IDE) in rat liver [3]. In vivo evidence suggested that the absence of hepatic IDE caused insulin resistance, higher blood glucose levels, and glucose intolerance through molecular mechanisms involving impaired hepatic insulin signaling and upregulation of gluconeogenic gene transcription [23]. Thus, CEACAM1 and IDE coordination regulate intracellular insulin trafficking and degradation to remove insulin from the circulation [15].

We do not yet understand the relative importance of insulin clearance mechanisms in the pathogenesis of obesity or other metabolic diseases. However, Mirsky is to be remembered as a giant of the field. 

## 3. Insulin Sensitivity and Insulin Secretion

**Figure 2:**
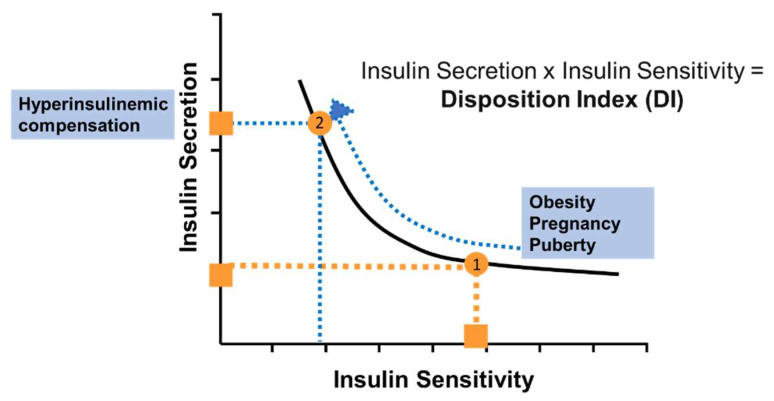
The “Law of Glucose Tolerance”. It is proposed that there is a hyperbolic relationship between insulin sensitivity and insulin release. Lower insulin sensitivity (insulin resistance) is associated with a greater insulin secretion, such that the product of sensitivity and secretion is a factor termed the “Disposition Index”. A lowering of insulin sensitivity (by increased adiposity, infection, pregnancy for example) would result in move of the orange dot (1) to the left (dotted orange line). However, it is proposed that associated with the reduction in insulin sensitivity would be an increase in beta-cell response. Thus, the trajectory would be represented as a simultaneous increase in insulin response, so the trajectory would be represented by a move along the blue curve to a point on the hyperbola, but on the upper left (2).

A vigorous debate ensued during the 1960s and beyond regarding the relative importance of deficiencies in insulin release versus insulin action in the pathogenesis of diabetes. Our group considered this conundrum and concluded that it was not appropriate to consider these two pathogenic factors in isolation, but it was important to consider them in relation to each other [24,25]. Based on a modeling approach, which we performed with Claudio Cobelli of the University of Padova, we hypothesized that there would be a stereotypical quantitative relationship between insulin sensitivity and insulin release which would be a hallmark of metabolic control. This relationship was predicted to be hyperbolic, such that the relation between insulin secretion and action could be defined by a hyperbolic curve (Figure 2). The equation defining this curve is the following:

(1) Secretion × Sensitivity = Disposition Index

The “Disposition Index” (“DI”) accounts for the behavior of the glucose-regulating system: any environmentally- or physiologically-caused reduction in insulin sensitivity (e.g., increased body weight [26], pregnancy [27], puberty [28], or aging [29]) would normally be compensated by an appropriate enhancement of insulin release. The ability of insulin release to increase in proportion to the increase in insulin resistance would be a measure of beta-cell health; thus, beta-cell function and insulin action can change, but the DI would remain constant.

**Figure 3:**
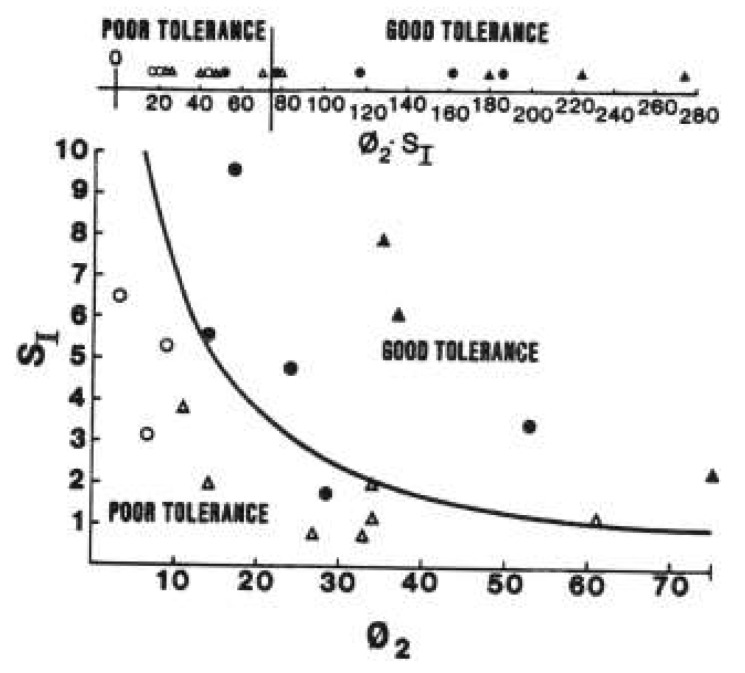
First publication of the hyperbolic relationship between beta-cell response (abscissa) and insulin sensitivity (ordinate). Subjects were segregated into those with poor glucose tolerance and good glucose tolerance. From Ref. [30].

We proposed that every individual could be characterized by a given DI which would determine the insulin response that would characterize their beta cell compensation for a given level of insulin resistance. By this concept, in response to any physiological change in insulin sensitivity (e.g., due to obesity, pregnancy, or puberty), an individual would mount an appropriate insulin secretion response, according to the disposition index equation. Working with Lawrence Phillips, we were the first to confirm this relationship in a group of human volunteers (Figure 3, [30]). 

The metabolic vicissitudes of life could then be defined in terms of the DI curve. The concept has now been generally accepted in the diabetes community; the number of publications utilizing this concept exceeds 1000.

**Figure 4:**
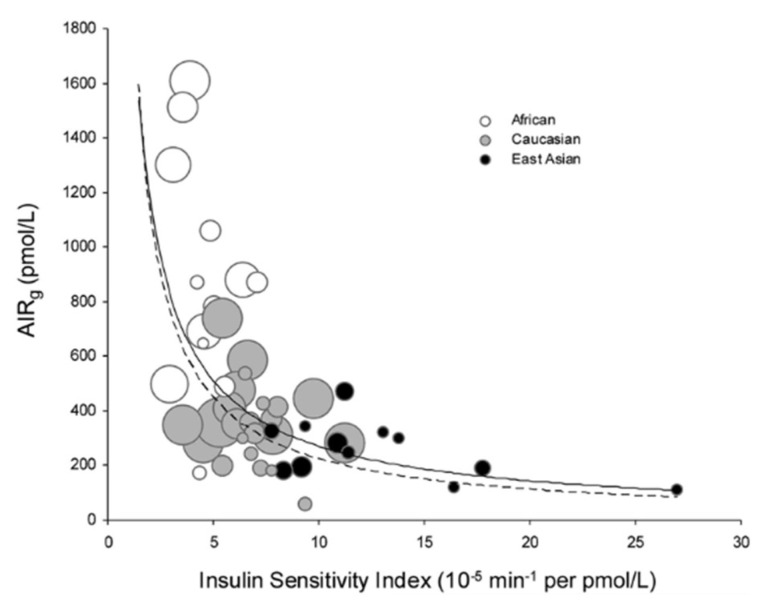
Meta-analysis by Kodama and colleagues of a large number of assessments of insulin sensitivity (abscissa) and insulin release (ordinate) in multiple cohorts of African, East Asian and Caucasian populations. Note that the best-fit hyperbola is a reasonable overall description of all participants. From Ref. [12].

It is not possible in this space to review all the multitude of uses to which the DI has been put. There is good evidence that the DI is heritable [31], with a heritability of approximately 0.7. The DI has been examined in different ethnic groups. In a remarkable publication, Kodama and Butte and colleagues performed a meta-analysis of various ethnic populations and demonstrated that, overall, the hyperbolic relationship applied for various ethnic groups [32]. Interestingly, Africans were more insulin resistant than Caucasians, while East Asians were more insulin sensitive. Of course, the beta-cell response was greater for Africans and less for East Asians; therefore, the DI curve was appropriate overall for all the ethnicities studied (Figure 4).

## 4. Possible Inadequacy of the Disposition Index Curve

What is missing from the DI curve? While insulin response and insulin sensitivity are expressed, insulin clearance is not included. We have already intimated, based upon the considerations due to Professor Mirsky, that insulin clearance may be an important, or even a critical factor in the determination of metabolic control. Therefore, to begin to understand the role of insulin clearance, we must return to a short consideration of the history of this concept.

## 5. Robert Turner’s Question

Professor Robert Turner was an icon of diabetes research. He and his colleagues at Oxford University performed the UKPDS, which examined the relationship between glycemia and risk of diabetic complications. Several decades ago, in a public meeting, Dr. Turner asked me, in particular, why evolution had chosen the somewhat bizarre vascular flow pattern in which effluent from the upper gastrointestinal tract (including the pancreatic beta-cells) flowed into the hepatic portal vein directly, rather than into the systemic circulation. At the time, I responded that I could not explain choices made by evolution or higher powers. However, as discussed below, it is possible that this flow pattern developed because physiological changes in hepatic insulin degradation might be a mechanism involved in the ability of the intact organism to compensate for insulin resistance by delivering a larger fraction of secreted insulin into the systemic circulation. Whether the latter phenomenon is beneficial or potentially harmful to those at risk for type 2 diabetes remains a mystery (see below).

## 6. Characteristics of Insulin Degradation

**Figure 5:**
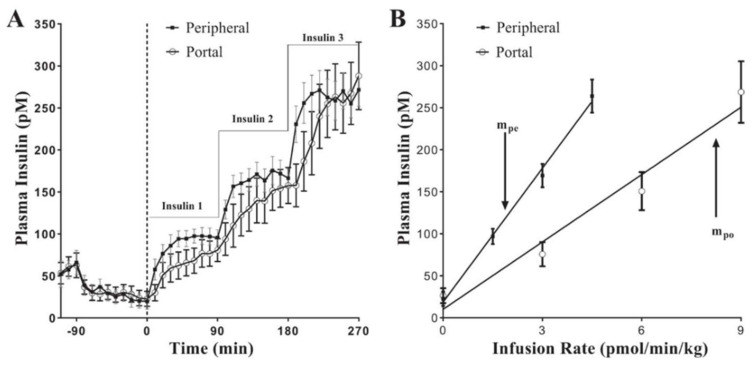
Euglycemic clamp experiments in which insulin was infused stepwise either systemically or into the portal vein of the liver. Infusion rates were chosen to attain approximately matched systemic insulin levels (see text; (**A**)). A plot of systemic insulin levels as a function of insulin infusion (**B**) yielded a lower slope with portal infusion due to first-pass liver insulin clearance. The ratio of the slopes in Figure 5B yielded a direct and accurate measure of first-pass hepatic clearance of insulin. From Ref. [33].

In our laboratory we have explored physiological control of insulin degradation. We developed an experimental model to measure insulin clearance by the liver directly. Experiments were conducted in the conscious canine model, in which we compared, under euglycemic clamp conditions, insulin infused systemically, or insulin infused directly into the hepatic portal vein [33]. Portal infusion rates during clamps were chosen to match resulting systemic insulin levels during systemic insulin infusion (Figure 5A). Clearly, intraportal infusion rates had to be about twice as great as systemic infusion rates, since about half the intraportally infused insulin was cleared by first-pass transition through the liver. Thus, the slope of the relationship between insulin infusion rate and resulting measured insulin was lower with intraportal infusion (Figure 5B); the ratio of these slopes yielded a direct assessment of the first-pass clearance of insulin. While average insulin clearance was substantial in normal animals, there was a surprising variance in basal insulin degradation among normal animals (22% to 77%) [33]. Despite that, high fat feeding further reduced mean first-pass hepatic clearance of insulin to 56% after six weeks, and 43% after twelve weeks of fat feeding [34]. These experiments demonstrated (1) that even in normal animals, first-pass insulin clearance by the liver was highly variable, and (2) that clearance of insulin by the liver was not constant in a single individual, but changeable with environmental conditions such as feeding of a high fat diet. Thus, it is necessary to think of first-pass hepatic insulin degradation as a dynamic variable and controllable parameter in the overall regulation of glycemic control.

Our experiments, and those of others, provide a potential answer to Robert Turner’s question cited above. The pattern of blood flow to the liver has emerged over time to provide a mechanism by which the intact organism can alter insulin clearance by the liver itself, and thus vary the delivery of insulin to the systemic tissues independent of changes in insulin secretion. Changes in degradation rate can and do occur under different environmental conditions. The question arises whether changes in liver insulin degradation are normal—and therefore protective of the organism—or whether they may be abnormal, possibly leading to conditions of metabolic risk, such as impaired glucose tolerance and/or diabetes itself.

## 7. Ethnic Differences in Insulin Clearance

We are very fortunate that Dr. Barbara Gower of the University of Alabama has allowed for measurement of first-pass hepatic insulin degradation in several cohorts of human patients in African American and European American ethnic groups. These groups exhibit different risks for metabolic disease, including type 2 diabetes, and it is of interest if there is a relationship between insulin degradation and diabetes risk. Working with Dr. Gower and with Dr. David Polidori of Janssen Research, we developed a modeling-based approach to measuring hepatic and extra-hepatic insulin clearance, utilizing the data from the intravenous glucose tolerance test Gower performed [35]. Reminiscent of our results in animals, again we discovered wide variance in insulin clearance rate among European American nondiabetic individuals; first-pass clearance varied from 10% to 80%. It is known that African Americans are more at risk for type 2 diabetes than European Americans; first-pass hepatic insulin clearance varied from near zero to 45% in that population. Thus, there is an apparent association between insulin clearance and risk for type 2 diabetes in different ethnic groups. The question arises as to whether this difference is of genetic origin. While this is unknown, it is of interest that first-pass hepatic clearance of insulin is lower in African American children (7–13 years of age) [36].

Thus, data from human volunteers confirms a wide variance in first-pass hepatic insulin extraction and supports the idea that lower insulin clearance is characteristic of individuals at increased risk for type 2 diabetes. What is unclear is whether lower insulin clearance is also important in the pathogenesis of type 2 diabetes.

## 8. Hypothesis: Role of Reduced Insulin Degradation in the Pathogenesis of Type 2 Diabetes

Those at risk for diabetes have a reduced degradation rate of insulin. One concept, put forth by DeFronzo and colleagues, is that the reduced clearance is part and parcel of the insulin resistance which characterizes obese subjects and those with impaired glucose tolerance and type 2 diabetes [37]. The available data are primarily associative. There is little support at this time that insulin resistance is causative for reduced clearance. Data which are available are based upon insulin levels which are observed during the euglycemic clamp procedure—the so-called “metabolic clearance rate” of insulin (MCR) is decreased in obesity, IGT, and type 2 diabetes. MCR is a reflection of overall insulin clearance, and thus does not inform regarding the particular role of liver insulin degradation, which we and others have previously shown to be physiologically regulated [15,34,38].

**Figure 6:**
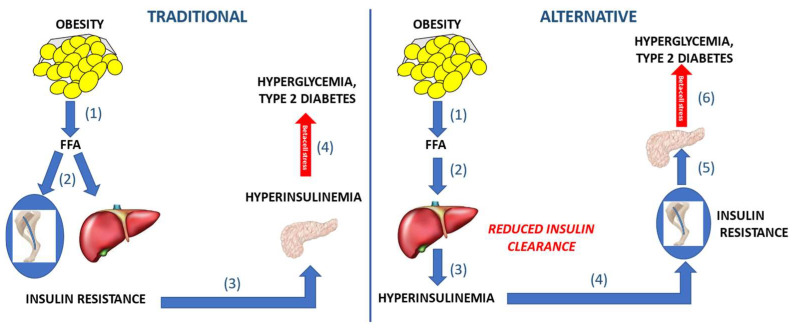
Two possible concepts of the pathogenesis of type 2 diabetes. Traditional concept (left panel): (1) Obesity causes increased flux of free fatty acids (FFA) flux which (2) results in insulin resistance at skeletal muscle and liver, (3) stimulating the beta-cells to release compensating insulin. (4) Elevated insulin compounds insulin resistance; when beta-cell compensation is inadequate, type 2 diabetes may ensue. Alternative concept (right panel): (1) Increased adiposity leads to release of FFA into circulation. (2) FFA are envisioned to reduce the clearance of insulin by the liver as a primary event in the pathogenesis. (3) Thus, lower degradation causes elevated insulin levels in blood. (4) Hyperinsulinemia exacerbates insulin resistance in skeletal muscle. (5) Insulin resistance necessitates a greater insulin secretory response in compensation; (6) ultimately beta-cell stress synergizes insulin resistance, causing type 2 diabetes.

We have suggested an alternative hypothesis—that reduced insulin clearance is a causative factor in risk for type 2 diabetes (Figure 6). By this concept, reduced first-pass hepatic degradation of insulin—whether caused by genetic or environmental factors—results in peripheral hyperinsulinemia. It is well documented that even experimentally elevated insulin can downregulate insulin action in several tissues (skeletal muscle, adipose tissue, brain, and the liver itself [39,40,41]. Thus, less insulin degradation would be associated with systemic insulin resistance. Insulin resistance could then stress the beta-cells, as greater insulin secretion would be necessary to maintain glucose tolerance. This scenario—reduced hepatic insulin clearance, peripheral hyperinsulinemia, insulin resistance and beta-cell stress—could characterize even those at risk for impaired glucose tolerance and type 2 diabetes in the prediabetic state. The question arises whether there are any longitudinal data which might support the hypothesis that lower insulin degradation is pathogenic for type 2 diabetes.

Shah and colleagues recently presented follow up results from a population of 570 Native Americans [42]. Over a 7.9-year period of observation, 32% of the cohort presented with type 2 diabetes. They reported that lower insulin clearance, measured by MCR (or MCRI, as used by Shah) during euglycemic clamps, was predictive of type 2 diabetes. The latter relationship was independent of other risk factors, including age, sex, body fat, heritage, or insulin disposal during clamps. They concluded that “lower MCRI [surrogate for insulin clearance] is related to an unfavourable metabolic phenotype and is associated with incident type 2 diabetes independent of established risk factors”.

The recent study of Shah and colleagues represents longitudinal data consistent with the hypothesis stated above—that lower insulin clearance is predictive of type 2 diabetes. Other longitudinal studies will be required to establish whether such a relationship can be observed in ethnic groups other than Native Americans. It will be of particular interest to examine whether hepatic insulin degradation per se will predict conversion of nondiabetic subjects to frank diabetes. The results of Shah et al. are particularly interesting because it is lower insulin clearance which predicted type 2 diabetes, confirmatory of the hypothesis we have put forth; however, this is not supportive of the concepts put forth by DeFronzo et al. that reduced insulin clearance is simply an overall reflection of insulin resistance, but not causative for conversion to type 2 diabetes. Additionally, it will be important to measure first-pass clearance of insulin by the liver directly, rather than metabolic clearance rate from clamps, to see if reduction in hepatic degradation of insulin is causative for diabetes. The intravenous glucose tolerance protocol, such as that used by Gower and Fernandez and colleagues [35,36], is an approach which measures liver clearance of the hormone and could be used in future longitudinal studies.

Of course, it is possible that the hyperinsulinemia which is part and parcel of the hypothesis stated above may be due to other causes, such as beta-cell hypersecretion [43]. While this is a possible contributor, we believe it may be less likely than a reduction in insulin clearance per se, as it is known that beta-cell defect, not oversecretion, is related to diabetes risk. It remains to be determined whether reduced insulin degradation by the liver or oversecretion by beta cells is more important in the development of type 2 diabetes. The comparison of these two possible mechanism represent an important area of research.

## 9. Robert Turner and I. Arthur Mirsky Revisited

We may now respond to Dr. Turner; the stereotypical blood flow pattern in the abdominal cavity allows for the insulin secreted by the beta-cells to immediately enter the hepatic circulation. The liver can then either allow the secreted hormone to bypass or can clear the secreted hormone from the circulation. This “choice” made by the liver depends upon genetic and environmental factors. Insulin is degraded in liver by the binding to hepatocyte receptors, internalization, and potential destruction by specific enzymes, including “insulin degrading enzyme (IDE)”, reminiscent if not identical to the “insulinase” discovered by Professor Mirsky. Thus, we have come full circle, identifying a physiological regulation system which is likely important under normal conditions to help maintain normal glucose tolerance, and which, if functionally abnormal due to genetics or other factors, can lead to diabetes. It is likely that our ability to address this system pharmacologically will be an independent avenue to possibly prevent and treat type 2 diabetes mellitus.

## Data Availability

Not applicable.
